# Effective mnemonic techniques for memorizing the peripheral nervous system: a focus on the brachial, lumbar, and sacral plexuses

**DOI:** 10.3389/fsurg.2025.1511451

**Published:** 2025-06-13

**Authors:** Zhai Tianjun, Quan Honglei, Chen Yeping, Feng Wei

**Affiliations:** ^1^ School of Acupuncture, Moxibustion and Tuina (School of Rehabilitation Medicine), Anhui University of Chinese Medicine, Hefei, China; ^2^ Chinese Medicine Department, The Second Rehabilitation Hospital of Shanghai, Shanghai, China; ^3^ Rehablitation Department, Air Force Medical University TANGDU Hospital, Shanghai, China

**Keywords:** mnemonic techniques, anatomical learning, educational strategies, medical education, peripheral nerve plexuses

## Abstract

**Background and aims:**

The complex anatomy of peripheral nerve plexuses, such as the brachial, lumbar, and sacral plexuses, poses significant challenges in medical education. Traditional educational methods often prove inadequate in facilitating the retention of detailed information about these nerve structures. This study aims to propose innovative mnemonic techniques to enhance memory retention and simplify the learning process for medical students and professionals.

**Methods:**

We developed and implemented various mnemonic techniques, including diagrams, rhyming mnemonics, and sequential methods, to facilitate the memorization of the peripheral nerve plexuses.

**Interpretation:**

The use of mnemonic techniques, including visual aids and structured learning strategies, can address the educational challenges posed by the intricate anatomy of peripheral nerve plexuses. These methods not only expedite the memorization of nerve branches but also elucidate their origins, enhancing diagnostic precision and therapeutic outcomes in clinical settings.

## Introduction

In foundational medical courses like human anatomy and systematic anatomy, the nervous system is frequently cited as one of the most challenging areas for students to master. Within this system, the peripheral nerve plexuses—composed of the anterior rami of spinal nerves, such as the brachial, lumbar, and sacral plexuses—pose significant memory challenges due to their complex structures, numerous branches, and extensive innervation territories. Moreover, without regular revision, the intricate details of these plexuses are prone to being forgotten.Peripheral nerve plexuses are intricate networks comprising various nerve fibers, playing a pivotal role in both sending commands from the brain and spinal cord to the body's extremities and relaying sensory feedback to the central nervous system (1). A profound understanding of these plexuses' structure and function is critical for medical students and professionals in related fields, as this knowledge directly influences the diagnosis and treatment of neurological disorders.

The importance of peripheral nerve plexuses extends beyond basic physiological functions; they are also vital in the body's response to disease or injury (2). Their complex architecture and the diversity of the involved nerves present substantial educational challenges. Each plexus, with its unique components and innervation areas, demands the memorization of extensive details, significantly increasing cognitive load. For instance, the brachial plexus, comprising multiple nerve roots from the cervical spine, forms a complex network that facilitates sensory and motor functions in the shoulders, arms, and hands. Similarly, the lumbar plexus, with its intricate design, controls movements and sensations in the lower limbs (3). Moreover, mastering peripheral nerve plexuses often requires strong spatial visualization skills, as students must mentally construct a three-dimensional model of these structures. Additionally, the isolation of this topic from daily applications or broader medical knowledge contributes to rapid forgetting of complex details. Confusion is further exacerbated by the similarities in the names and pathways of different plexuses.

Numerous studies have highlighted a trend: diversified teaching methods can enhance learning outcomes and better prepare students for practical applications. For instance, Simons explored teaching methods in anatomy education. Their findings support an integrated learning approach that combines traditional methods with innovative tools to enhance understanding and retention, a strategy that can be effectively adapted across various professional fields (4). Roxburgh and Evans offer a broader perspective on assessing anatomy education, proposing that design thinking can significantly enhance the educational process by focusing on how students engage with and understand anatomical concepts (5). These studies collectively advocate for a multifaceted approach to anatomy education, where traditional methods are complemented with innovative technologies and teaching strategies tailored to the specific needs of various fields.

To enhance the retention of these complex structures, the use of diagrams, mnemonic devices, and pattern recognition is recommended. Techniques like employing rhymes or rhythmic mnemonics to remember various nerve branches have proven effective (6). This article describes several conceptual approaches for aiding in the memorization of the brachial, lumbar, and sacral plexuses, utilizing diagrams, mnemonic devices, and sequential learning strategies.

## Memory techniques for the brachial plexus: the diagram method

The brachial plexus is an intricate component of the peripheral nervous system, critical for neurosurgeons, pain management specialists, rehabilitation physicians, and orthopedic doctors diagnosing and treating upper limb conditions. The plethora of branches, such as the radial, axillary, ulnar, musculocutaneous, and median nerves, along with others including the dorsal scapular, suprascapular, long thoracic, lateral pectoral, medial pectoral, subscapular, medial brachial cutaneous, and medial antebrachial cutaneous nerves, creates substantial challenges in clinical memorization, often leading to confusion regarding their nerve root origins (7).

To address these challenges, simple and effective memory techniques are invaluable. The diagram method, in particular, facilitates quick recall of nerve branches and their origins. By visually mapping these nerves, learners can foster an intuitive understanding of their branching and interconnections, thus demystifying a complex anatomical structure for easier study and clinical application. This method not only accelerates the memorization of nerve branches but also enhances clarity regarding their roots, benefiting both academic learning and clinical practice.

**Figure 1:**
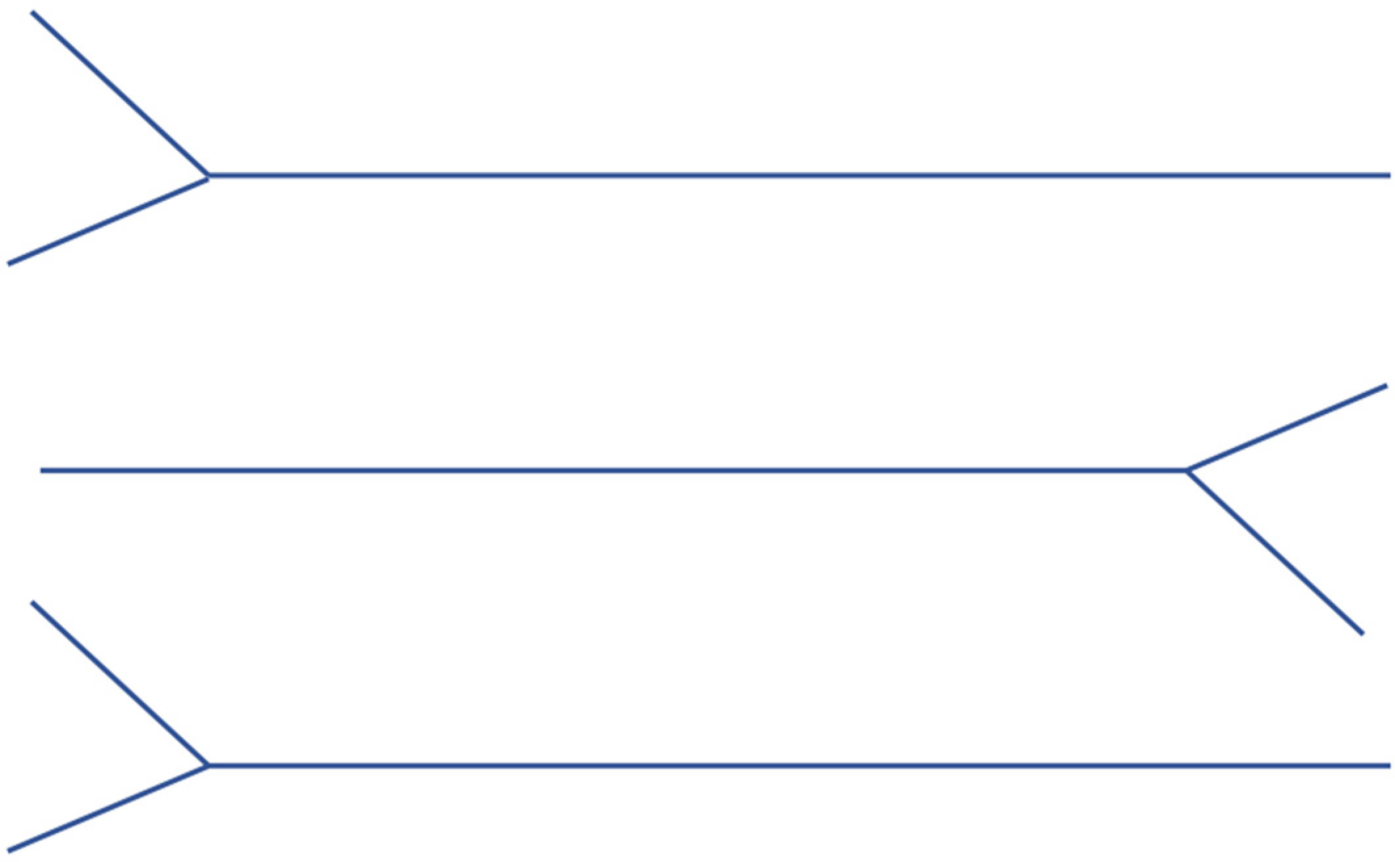
Step 1 of drawing the brachial Plexus.

(1) Start by drawing the upper, middle, and lower trunks of the brachial plexus as simple “Y” shapes, establishing the foundational structure from which all branches will extend, see Figure 1.

**Figure 2:**
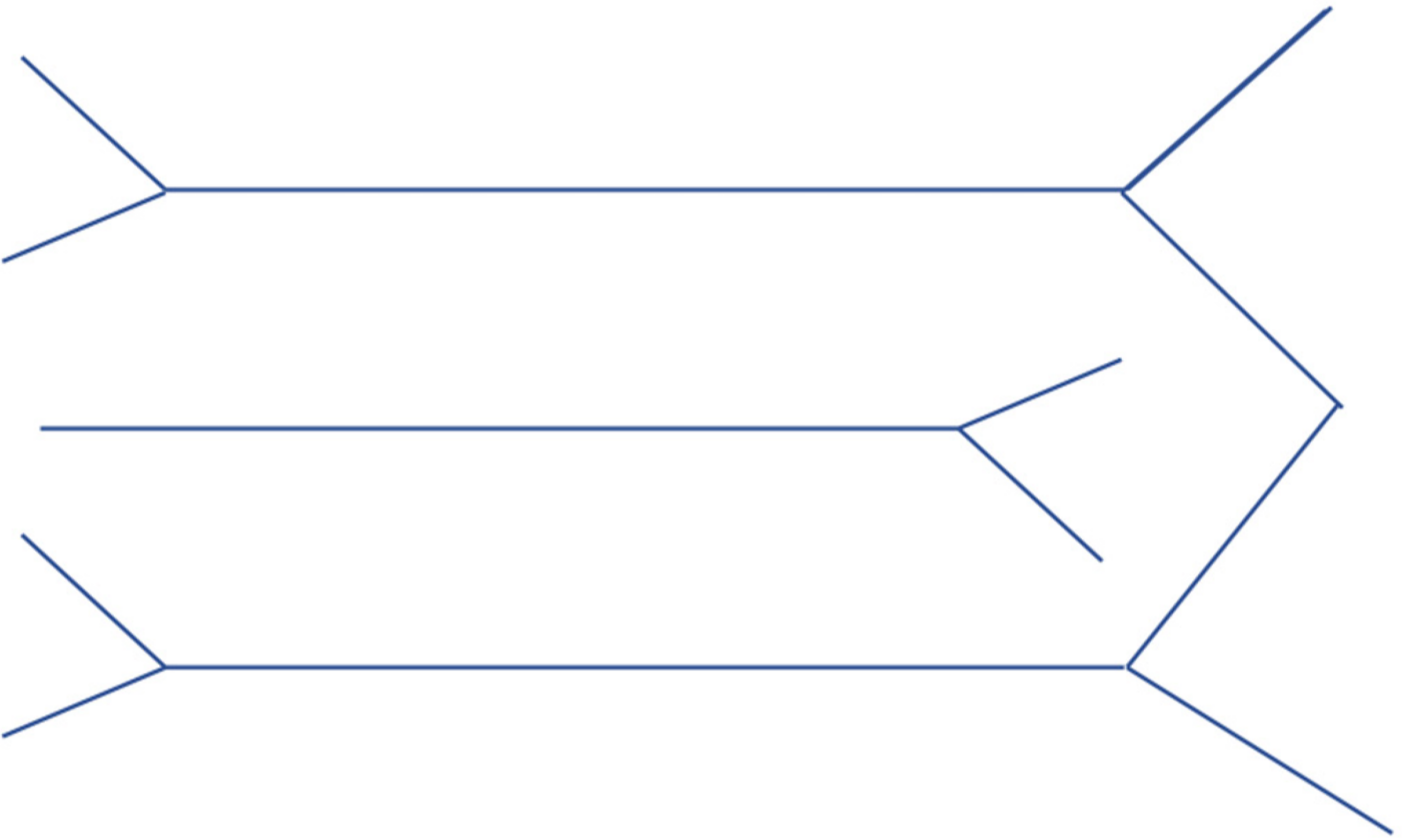
Step 2 of drawing the brachial Plexus.

At the ends of the first and third “Y”, add a “W” shape to represent the divisions of the brachial plexus, see Figure 2.

**Figure 3:**
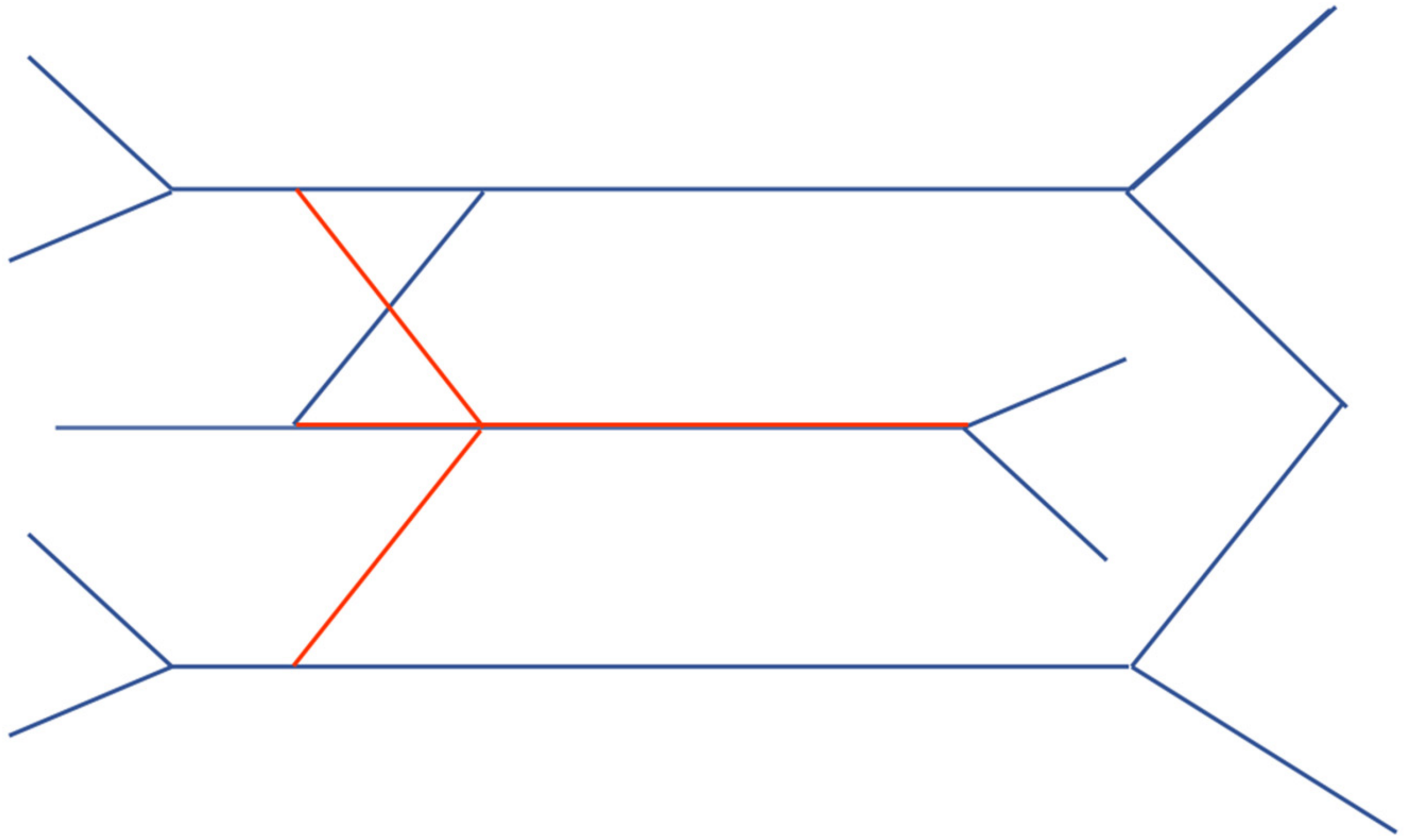
Step 3 of drawing the brachial Plexus.

Illustrate the anterior and posterior divisions for each of the upper, middle, and lower trunks (Blue lines represent anterior division, while red lines are posterior division), further defining the pathways of nerve branches, see Figure 3.

**Figure 4:**
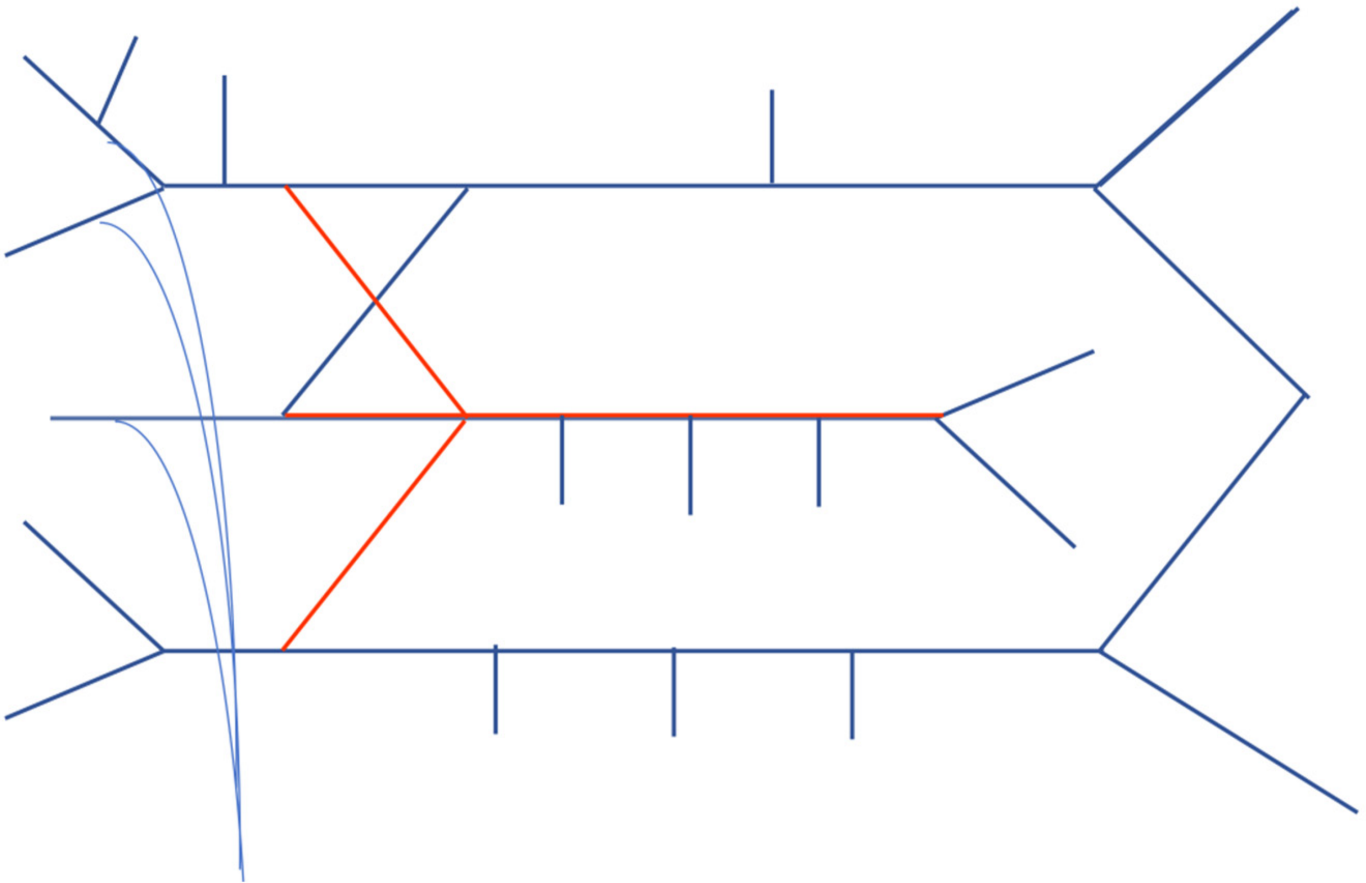
Step 4 of drawing the brachial Plexus.

Detail the remaining branches, notably including those from the C5 nerve root, branches from the upper trunk, and branches formed by the C5, C6, and C7 nerve roots, see Figure 4.

**Figure 5:**
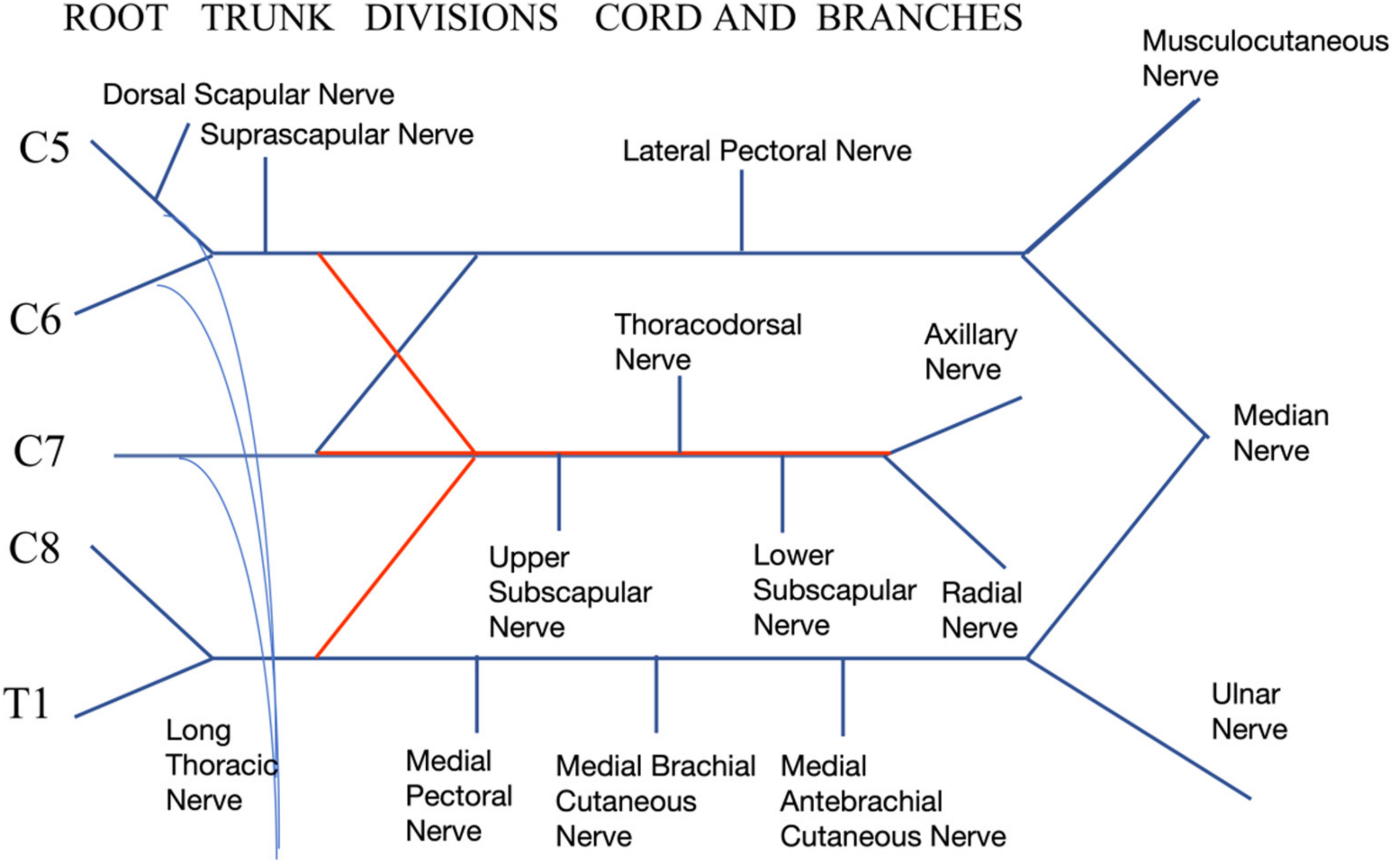
Step 5 of drawing the brachial Plexus.

Label each nerve branch and enhance understanding by adding annotations to each part of the diagram. This step is crucial for effective memorization and understanding the function and area of innervation for each branch, see Figure 5.

The brachial plexus consists of five structural components: roots, trunks, divisions, cords, and branches. Initially, the brachial plexus is formed by the merging of spinal nerve roots into trunks: the C5 and C6 roots create the upper trunk, C7 forms the middle trunk, and C8 and T1 combine to establish the lower trunk, defining the roots and trunks segments.

Each of the upper, middle, and lower trunks splits into anterior and posterior divisions. All posterior divisions converge to form the posterior cord, whereas the anterior divisions of the upper and middle trunks merge to create the lateral cord, and the anterior division from the lower trunk forms the medial cord.

Various branches emerge from these cords. Notably, branches originating above the clavicle, such as the dorsal scapular nerve (C5), suprascapular nerve (C5 and C6), and long thoracic nerve (C5, C6, and C7), are classified as supraclavicular branches. Conversely, branches passing through the costoclavicular space to become infraclavicular branches include the lateral pectoral, superior subscapular, thoracodorsal, inferior subscapular, medial pectoral, medial brachial cutaneous, medial antebrachial cutaneous nerves, along with the axillary, radial, musculocutaneous, median, and ulnar nerves.

Mapping the brachial plexus identifies five predominant nerve branches: the musculocutaneous nerve from the lateral cord, the ulnar nerve from the medial cord, the median nerve formed by fibers from both the medial and lateral cords, and the axillary and radial nerves from the posterior cord. Specific branches such as the dorsal scapular nerve, which innervates the levator scapulae and rhomboid muscles; the suprascapular nerve, which supplies the supraspinatus and infraspinatus muscles along with the shoulder joint capsule; and the long thoracic nerve, which innervates the serratus anterior muscle, originate from combinations of the C5 to C7 nerve roots.

Understanding the brachial plexus's mapping enables students to identify the root origins of each nerve, such as the ulnar nerve deriving from the C8 and T1 spinal nerve roots, and the median nerve from the C5 to T1 roots. This knowledge also aids in understanding common clinical symptoms, such as medial scapular pain in brachial plexus entrapment syndromes due to compression of the dorsal scapular nerve, or muscle atrophy in frozen shoulder syndrome, where the axillary and suprascapular nerves play critical roles in innervating the shoulder capsule and rotator cuff muscles (8).

### Memory techniques for the lumbar plexus: mnemonic technique

**Figure 6:**
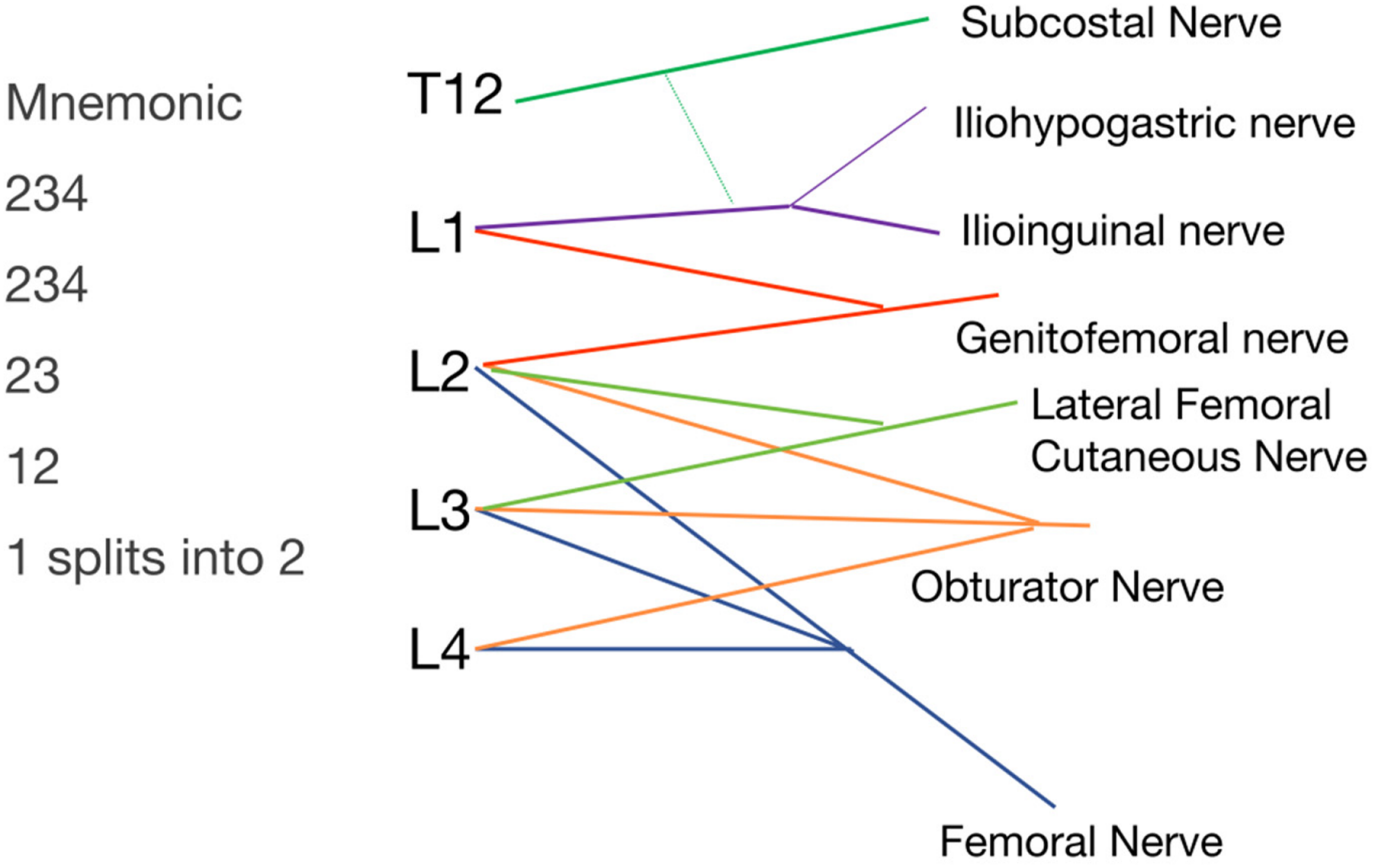
Lumbar plexus nerves and diagram.

The lumbar plexus, formed by the anterior rami of spinal nerves T12-L4, comprises six primary branches: the iliohypogastric nerve, ilioinguinal nerve, genitofemoral nerve, lateral femoral cutaneous nerve, obturator nerve, and femoral nerve. Notably, the iliohypogastric, ilioinguinal, and lateral femoral cutaneous nerves travel along the lateral edge of the iliopsoas muscle, the genitofemoral nerve runs anterior to the iliopsoas, and the obturator nerve follows the medial edge of this muscle (9). A useful mnemonic for remembering the lumbar plexus structure is “234, 234, 23, 12, 1 splits into 2,” representing the convergence of nerve roots L2, L3, L4 to form various nerves, culminating in six distinct nerves, see Figure 6.

For effective integration of the mnemonic with specific nerves, a comprehensive understanding of the dermatomal distribution is essential. The L1 nerve root dermatome is situated in the inguinal region, L2 in the upper thigh, L3 on the internal and lateral thigh, and L4 covers the area in front of the knee and the medial side of the lower leg, extending beyond the knee (10). Consequently, the first “234” in the mnemonic corresponds to the femoral nerve, which innervates the anterior thigh, knee area, quadriceps, pectineus, and hip joint capsule. The second “234” is associated with the obturator nerve, responsible for the medial thigh and upper part of the popliteal fossa, including the adductors and hip joint capsule. The “23” is linked to the lateral femoral cutaneous nerve, serving the lateral thigh. The “12” matches with the genitofemoral nerve, branching into genital and femoral branches that cover the upper inner thigh and genital area. The “1 splits into 2” refers to the iliohypogastric and ilioinguinal nerves, which also innervate certain lower abdominal muscles; T12 typically forms the subcostal nerve, occasionally contributing to the iliohypogastric and ilioinguinal nerves.

This mnemonic technique, when integrated with knowledge of dermatomal distributions, enables students to swiftly ascertain the origins of nerves for each branch of the lumbar plexus and facilitates understanding of various clinical symptoms. For instance, a herniation of the L3, L4 discs compressing the L4 root may lead to diminished knee extension, and strength in the tibialis anterior (11). Compression fractures at T12 frequently manifest as lower abdominal pain and spasms in the abdominal muscles (12). Moreover, simultaneous groin pain and anterior knee pain may suggest mild compression of the lumbar plexus as it traverses the iliopsoas muscle (13). Accurately grasping this concept is crucial for the effective diagnosis and management of conditions associated with lumbar plexus pathology.

### Memory techniques for the sacral plexus: sequential method

The sacral plexus, comprising the anterior rami of spinal nerves from L4 to Co, includes critical nerves such as the superior gluteal, inferior gluteal, posterior femoral cutaneous, pudendal, and sciatic nerves (14). The superior gluteal nerve emerges from L4, L5, and S1 nerve roots, while the inferior gluteal nerve is derived from L5, S1, and S2. The posterior femoral cutaneous nerve, which also gives rise to the inferior cluneal nerves, originates from S1, S2, and S3. The pudendal nerve originates from S2, S3, and S4. These nerves generally involve combinations of three nerve roots from proximal to distal sections.

**Figure 7:**
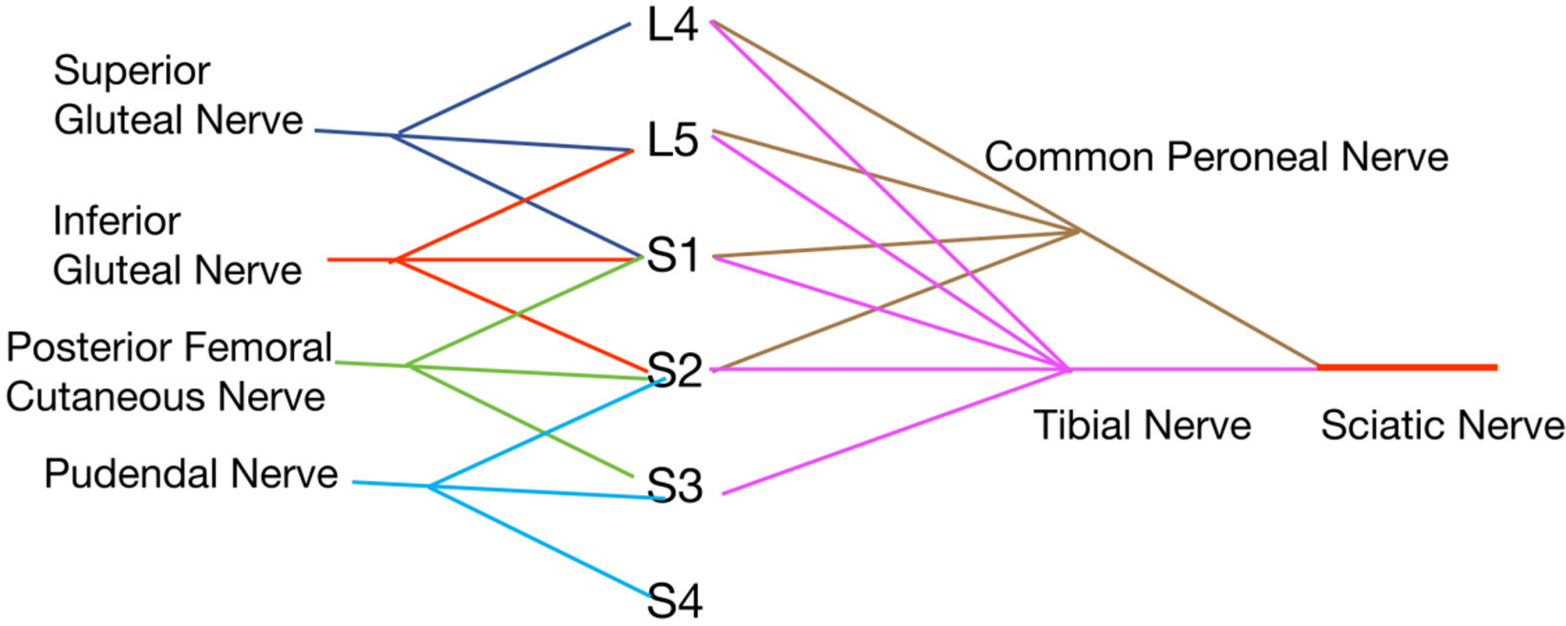
Sacral plexus nerves and diagram.

The sciatic nerve, which divides into the tibial and common peroneal nerves, draws from L4, L5, S1, S2, and S3 nerve roots. Notably, the common peroneal nerve, responsible for innervating the dorsum of the foot, includes nerve roots up to S2, while the tibial nerve, innervating the sole, extends to S3. This configuration establishes the sciatic nerve with root origins from L4 to S3, see Figure 7.

Each nerve within the sacral plexus follows a specific anatomical path and innervation pattern:

• The superior gluteal nerve, consisting of L4, L5, and S1, passes above the piriformis muscle to innervate the gluteus medius, gluteus minimus, and tensor fasciae latae muscles.
• The inferior gluteal nerve, comprised of L5, S1, and S2, runs below the piriformis, innervating the gluteus maximus.
• The posterior femoral cutaneous nerve, originating from S1 to S3, exits below the piriformis, providing innervation to the skin of the posterior thigh and the lower buttock area.
• The pudendal nerve, emerging from S2 to S4, innervates parts of the pelvic floor muscles and supplies sensory innervation to the genital area.
• The sciatic nerve, composed of L4 to S3, descends through the greater sciatic foramen, splitting into the tibial and common peroneal nerves, each targeting distinct regions.

Understanding the sequential arrangement and specific contributions of nerve roots to each nerve within the sacral plexus enables students to effectively correlate clinical symptoms with related pathologies. For example, a disc herniation impinging on these nerve roots can cause distinct lower limb and pelvic symptoms, requiring precise anatomical knowledge for effective management. This structured approach simplifies both learning and applying sacral plexus anatomy in clinical practice.

## Discussion

Peripheral nerve plexuses, such as the brachial, lumbar, and sacral plexuses, play a vital role in diagnosing and managing various pain conditions. A deep understanding of these plexuses is crucial for accurate diagnostics, precise nerve block techniques, and effective management of chronic pain. This article describes the use of innovative mnemonic techniques aimed at facilitating the memorization of peripheral nervous system anatomy, with a focus on the brachial, lumbar, and sacral plexuses. By incorporating visual aids and structured learning strategies, these mnemonic approaches serve as effective educational tools. While promising for enhancing anatomical knowledge among students and professionals, these methods should be considered exploratory and their effectiveness in clinical practice requires further validation.

### Traditional vs. modern teaching methods in anatomy education

In the discussion of anatomy education methods, cadaver labs and surgical experience have long been considered foundational, offering real tactile feedback and spatial awareness crucial for surgical training. However, the accessibility and cost of these methods limit their widespread use. In recent years, virtual reality(VR) technology has emerged as an innovative teaching choice due to its interactivity and repeatability, though it still lacks in providing tactile feedback (15). Mnemonic techniques, serving as supplementary tools, help students preliminarily establish a cognitive framework of complex anatomical structures before engaging in more in-depth practical learning. By integrating mnemonic techniques with traditional cadaver lab teaching, we can create a multifaceted educational environment aimed at enhancing students' learning experiences and educational outcomes, maximizing the efficiency of educational resources, ensuring that students comprehensively grasp anatomical knowledge and apply it in real medical practices. This integrated educational strategy not only strengthens the connection between theoretical learning and practical application but also provides a new direction for the future development of medical education.

### Future directions and research opportunities

Looking forward, our research could evolve in several promising directions:We plan to conduct longitudinal studies to assess the enduring impact of mnemonic techniques on memory retention. This will help determine the duration of effectiveness and the necessity for periodic reviews within medical curricula.Combining mnemonic techniques with emerging educational technologies, such as Augmented Reality (AR) and VR, presents a promising avenue for creating more immersive and interactive learning experiences. This integration is likely to enhance spatial understanding of anatomical structures significantly.

We aim to investigate how mnemonic techniques prepare medical professionals for real-world clinical scenarios. This involves tracking the performance of practitioners who utilize these techniques in diagnosing and treating patients, particularly in complex cases involving the peripheral nervous system.Our research specifically targets the complex structures of the peripheral nerve plexuses, namely the brachial, lumbar, and sacral plexuses. These regions are notoriously difficult to master due to their intricate structures and extensive innervation. Our mnemonic techniques are specifically designed to address these complexities, providing students and professionals with a more effective tool to master these key areas, laying a solid foundation for future related clinical work.

### The application of this mnemonic techniques in clinical work

Mnemonic techniques have been widely used in medical education to improve the retention and recall of complex information, especially in learning the anatomy of peripheral nerve plexuses. These methods provide significant advantages in multiple areas of medical practice.

### Importance of mnemonic methods for memorizing nerve names

For medical students and healthcare professionals, memorizing complex nerve plexuses such as the brachial, lumbar, and sacral plexuses often presents a significant challenge. Mnemonic techniques simplify this process by transforming abstract anatomical information into easy-to-remember visual and textual patterns. For instance, when considering the brachial plexus, the use of drawing techniques not only clearly displays the origins of each nerve root and the structural divisions of the plexus (roots, trunks, divisions, cords, branches) but also enhances understanding and memorization through visualization. Furthermore, repeatedly sketching these structures not only boosts the learner's drawing skills but also deepens the memory of these anatomical details through continuous repetition, thus enhancing the accuracy and longevity of memory.

This method of drawing mnemonics can be complemented with other educational tools such as three-dimensional models and interactive software to further enhance the learning experience. Three-dimensional models offer a comprehensive perspective on the spatial layout of nerve plexuses, while interactive software allows students to practically manipulate and test their knowledge through virtual surgeries or simulated tests. This multisensory approach to learning enables students not only to gain a deeper understanding of the functions and clinical significance of nerve plexuses but also to recall and apply this knowledge more rapidly in real clinical scenarios.

In medical practice, these mnemonic and drawing techniques are crucial for quickly and accurately identifying specific nerve injuries or pathological conditions. For example, emergency physicians can rapidly diagnose acute pain or functional impairments caused by specific nerve roots by quickly sketching or reviewing the structure of the brachial plexus. Thus, these mnemonic methods not only improve the efficiency of anatomical learning but also directly support the decision-making capabilities of healthcare professionals in urgent and high-pressure environments.

### Accurate diagnostics: correspondence between symptoms and imaging

Discrepancies between patient symptoms and imaging findings often lead to misdiagnoses, emphasizing the importance of a thorough understanding of nerve root origins to align clinical manifestations with anatomical evidence. Furthermore, the technique of nerve blockade, fundamental in pain management, relies heavily on precise identification of nerve roots to ensure effective and safe treatment.

Understanding the specific origins of peripheral nerve trunks and their nerve roots is crucial for physicians to make accurate clinical diagnoses, especially when dealing with complex neurological conditions. For example, disc herniation is commonly seen in the lumbar spine, and imaging might show a protrusion at the L4/L5 level. In such cases, patients typically experience pain and numbness on the lateral side of the lower limb, primarily due to the involvement of the L5 nerve root. However, if a patient's symptoms manifest on the inner thigh near the groin, this might involve the higher L1 and L2 nerve roots (16). These symptoms do not match the imaging findings, hence such patients often cannot be diagnosed with disc herniation. Understanding these details allows physicians to go beyond standard imaging diagnoses to more accurately interpret symptoms and provide treatment. Pain symptoms in the shoulder and neck are also often associated with specific nerve roots. For instance, a C4/C5 disc protrusion might affect the C5 nerve root, leading to pain along the medial edge of the scapula due to injury to the dorsal scapular nerve originating from the C5 nerve root (17). Thus, if a patient with medial edge of the scapula pain also shows C4/C5 disc protrusion, the symptoms align with the imaging findings, and it can be considered from a cervical spine disorder perspective (corresponding relationships, see Table 1). If the symptoms do not match the imaging findings, it is necessary to consider dorsal scapular nerve involvement or other biomechanical factors. However, if pain along the medial edge of the scapula is accompanied by numbness in some fingers, these symptoms might indicate compression of the brachial plexus by the scalene muscles at the thoracic outlet, rather than purely a cervical spine issue. The accuracy of such a diagnosis relies not only on imaging results but also on the physician's deep understanding of anatomical structures and nerve distributions.

**Table 1:** Corresponding relationships between nerve root and innervation areas.

Nerve Root	Innervation areas	Disc herniation most commonly affects segments
C5	Medial edge of the scapula, lateral epicondyle of the humerus	C4/C5
C6	Scapula, thumb	C5/C6
C7	Middle finger	C6/C7
C8	Little finger	C7/T1
T1	Medial epicondyle of the humerus	T1/T2

Therefore, physicians need comprehensive anatomical knowledge and an understanding of the specific symptoms that different nerve roots may cause. This knowledge not only helps physicians identify abnormalities during routine neurological examinations but also in selecting the most appropriate points for nerve blockades or other neurological interventions. Additionally, this knowledge is indispensable in detailed pre-surgical planning and postoperative management to ensure that treatment measures are both safe and effective.

### Precision in pain localization and nerve blockades

Peripheral nerve plexuses such as the brachial, lumbar, and sacral plexuses are key targets for regional nerve blockades. Nerve blockade involves injecting local anesthetics to interrupt nerve signals, thus alleviating pain in specific areas (18). For example, brachial plexus blocks are commonly used for pain control following arm and shoulder surgeries, as well as managing acute trauma and chronic pain conditions (19). Pain along the medial edge of the scapula may indicate compression of the dorsal scapular nerve, treatable with a nerve block at the C5 nerve root (20); similarly, pain in the scapular area might suggest irritation of the suprascapular nerve, addressable with a block at the C6 nerve root (21); and numbness in the middle thumb can be treated at the C7 nerve root.

The success of nerve blockades largely depends on an accurate understanding of nerve root distributions. For instance, knowledge of specific nerve roots in the lumbar plexus is vital for managing leg pain or surgeries because it includes major nerves like the femoral nerve (L2, L3, L4) and the obturator nerve (L2, L3, L4), which respectively innervate the anterior and medial parts of the thigh; thus, nerve root blockades for knee pain often focus on the L2, L3, and L4 nerve root areas. Similarly, sacral plexus blocks, especially sciatic nerve blocks, are crucial for managing pain in the lower limbs following hip and knee surgeries, requiring attention to the L4, L5, and S1–S3 nerve roots from a nerve root perspective. We will present two examples of the application of the mnemonic methods described in this paper in clinical treatment.

Case 1. A 35-year-old male presented to the clinic with acute right shoulder pain following a minor sports injury. Initial physical examination and imaging suggested a potential brachial plexus injury. The patient reported severe pain and restricted range of motion in the shoulder, accompanied by sensory deficits along the distributions of the axillary and musculocutaneous nerves. The attending orthopedic specialist utilized mnemonic strategies to promptly review the involved nerve anatomy. By employing the Diagram Method for the Brachial Plexus, the physician was able to swiftly correlate the patient's symptoms with potential nerve root injuries at C5 and C6, which constitute the upper trunk of the brachial plexus. This mnemonic-based understanding guided the decision to perform targeted nerve conduction studies, which confirmed neuropraxia at the upper trunk of the brachial plexus. This precise diagnosis facilitated a targeted therapeutic approach. The patient received corticosteroid injections aimed at the C5 and C6 nerve roots and participated in physical therapy that included nerve gliding exercises to enhance nerve recovery. The mnemonic technique enabled the physician to rapidly establish a diagnosis and initiate appropriate treatment.

Case 2. A 42-year-old female presented to the clinic with persistent pain on the lateral side of her right thigh, described as burning and tingling, exacerbated by prolonged standing or walking. The patient reported a sedentary occupation that involves long periods of sitting. The attending physician initially suspected compression of the lateral femoral cutaneous nerve as the cause of the pain. However, after two unsuccessful attempts at nerve block at the entrapment points of the lateral femoral cutaneous nerve, the patient reported no relief.At this point, the physician referred to the mnemonic technique for the lumbar plexus, focusing on the “23 nerve roots” mnemonic, which addresses the L2 and L3 nerve roots. Subsequent nerve root blocks administered at L2 and L3 significantly alleviated the patient's symptoms. This case highlights the importance of accurately diagnosing and targeting specific nerve roots in managing neuropathic pain, underscoring the utility of mnemonic techniques in clinical practice.

Accurate identification and understanding of these structures are essential for effective nerve blockade.

### Chronic pain management

Chronic pain symptoms, such as those in the shoulder girdle or lower limbs, are often closely linked to abnormalities in peripheral nerve plexuses. For physicians, a deep understanding of these complex neural structures is crucial both for diagnosing the source of pain and for selecting the most appropriate treatment options. For example, chronic pain in the shoulder girdle is typically associated with brachial plexus dysfunction, which can be treated with specialized neuromodulation techniques such as electrical stimulation or local anesthetic injections. These methods are aimed not only at alleviating pain but also at significantly restoring the function of the damaged nerves (22).

In managing chronic pain in the lower limbs, precise knowledge of the specific nerve distributions in the lumbar and sacral plexuses is particularly important. This understanding is critical for determining suitable treatment plans. For instance, pain in the posterior aspect of the thigh may be considered from a neurological perspective to be due to damage in the sciatic nerve (L4, L5, S1–S3) affecting its motor fibers, or it could be due to damage in the posterior cutaneous nerve of the thigh (S1–S3), causing pain in its dermatomal skin area. With knowledge of the specific nerve root origins, treatments such as spinal traction or deep tissue massage can effectively alleviate nerve root pressure (23), thereby reducing pain. In some cases, the use of thermotherapy, cryotherapy, or ultrasound treatment can further relieve pain and promote tissue healing.

Additionally, employing nerve blockade techniques, especially targeted treatment of specific nerve roots, can provide direct and rapid pain relief for patients. For example, a block of the sciatic nerve can provide immediate pain control following buttock and lower limb surgeries. Integrating physical therapies such as acupuncture, transcutaneous electrical nerve stimulation (TENS), and tailored exercise programs can further assist patients in managing their symptoms and promoting nerve function recovery (24).

This article has presented a variety of mnemonic techniques intended to aid in the memorization of complex anatomical structures within the peripheral nervous system, specifically targeting the brachial, lumbar, and sacral plexuses. While these methods, which include the use of diagrams, rhyming mnemonics, and sequential learning strategies, hold promise for enhancing educational outcomes, it is important to note that this is a preliminary proposal. The effectiveness of these techniques has not been empirically demonstrated due to the lack of experimental design and statistical analysis in this study. Consequently, while the described methods are innovative and potentially valuable educational tools, they should currently be considered as conceptual approaches. Future research should aim to provide empirical evidence through rigorously designed experiments and statistical evaluations to substantiate the efficacy of these mnemonic techniques in medical education. Until such data is available, these techniques should be integrated cautiously into educational practices, with an understanding of their theoretical rather than proven practical benefits.

## Data Availability

The original contributions presented in the study are included in the article/Supplementary Material, further inquiries can be directed to the corresponding authors.
